# Meckel’s diverticulum with intraperitoneal hemorrhage in a child detected with screening laparoscopy: a case report

**DOI:** 10.1186/s40792-021-01347-9

**Published:** 2021-12-20

**Authors:** Kazuki Wakizaka, Lee Wee Khor, Kazuya Annen, Tsuyoshi Fukushima, Mitsuko Furuya, Akinobu Taketomi

**Affiliations:** 1 Department of Surgery, Chitose City Hospital, 2-2-1, Hokko, Chitose, Hokkaido 066-8550 Japan; 2 Pathology Center, GeneticLab Co., Ltd., 28-196, N9-W15, Chuo-ku, Sapporo, Hokkaido 060-0009 Japan; 3 Department of Gastroenterological Surgery I, Hokkaido University Graduate School of Medicine, N15-W7, Kita-ku, Sapporo, Hokkaido 060-8638 Japan

**Keywords:** Meckel’s diverticulum, Intraperitoneal hemorrhage, Laparoscopic surgery, Pediatric, Case report

## Abstract

**Background:**

The most common presentation of symptomatic Meckel’s diverticulum (MD) are intestinal obstruction, gastrointestinal hemorrhage, and inflammation of the MD with or without perforation. Intraperitoneal hemorrhage because of MD is extremely rare. We report a case of MD with intraperitoneal hemorrhage in a child detected with screening laparoscopy.

**Case presentation:**

An 11-year-old girl presented to another hospital with lower abdominal pain and vomiting that lasted for 2 days. Acute appendicitis was suspected, and she was referred to our department. Abdominal enhanced computed tomography showed an abscess in the lower abdomen with ascites in the pelvis. She was diagnosed with a localized intra-abdominal abscess and the decision was made to treat with antibiotics. However, her abdominal pain worsened, with abdominal distension, tenderness and guarding. She was diagnosed with panperitonitis and the decision was made for surgery 5 h after admission. During surgery, laparoscopic observation from the umbilical region revealed 200 ml of fresh blood throughout the peritoneal cavity, originating from the mesentery of the ileum. MD was observed with bleeding from the surrounding mesentery. Small bowel resection was performed, and the patient was discharged on the 5th postoperative day. Pathological findings revealed an MD containing ectopic gastric mucosa and small intestinal ulcer perforation at the base of the MD.

**Conclusions:**

We report an extremely rare case of an MD with intraperitoneal hemorrhage in a child. In pediatric cases, it is possible that perforation with ectopic gastric mucosa may cause massive bleeding because of rupture of the surrounding mesenteric blood vessels.

## Background

A Meckel’s diverticulum (MD) is congenital diverticulum on the ileum resulting from incomplete atrophy of the vitelline duct in the embryo [1]. Its prevalence is reported to between 0.3 and 2.9% in the general population, and most cases are asymptomatic throughout life [1]. The most common presentation of symptomatic MD are intestinal obstruction, gastrointestinal hemorrhage, and inflammation of the MD with or without perforation [1]. Intraperitoneal hemorrhage resulting from an MD is extremely rare. We report a case of MD with intraperitoneal hemorrhage in a child detected with screening laparoscopy.

## Case presentation

**Fig. 1:**
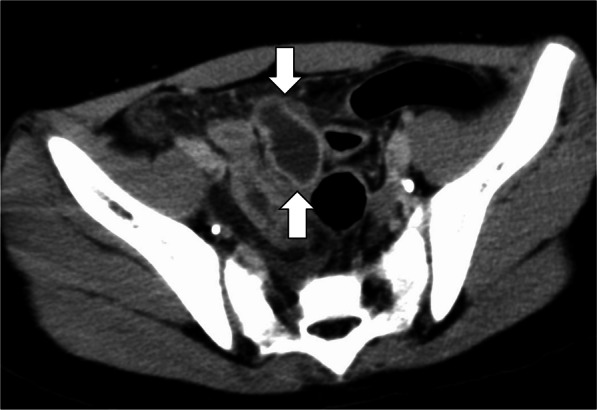
Abdominal image. Preoperative abdominal enhanced computed tomography showing an abscess in the lower abdomen (arrow), which was ultimately diagnosed as Meckel’s diverticulum

**Fig. 2:**
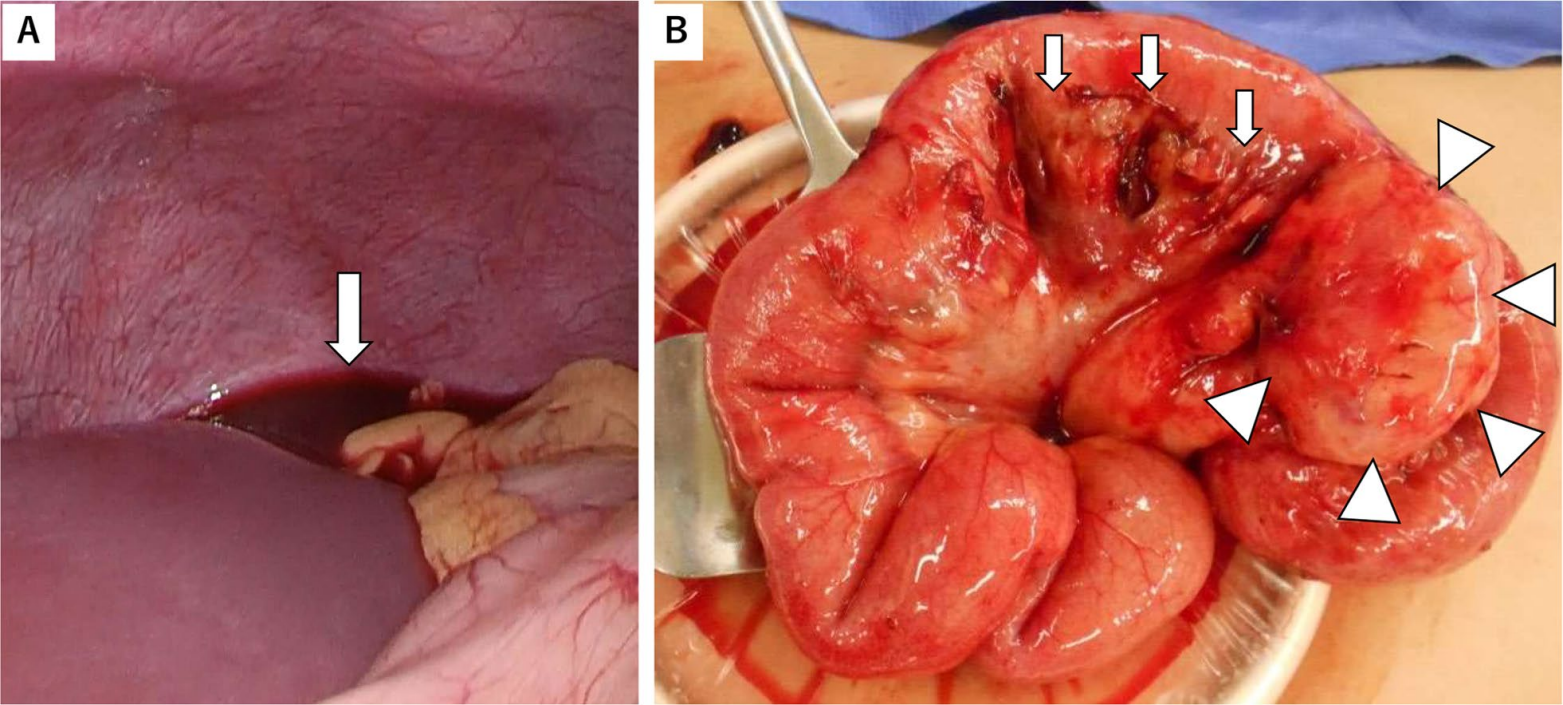
Intraabdominal findings. **A** Laparoscopic observation revealed a total of 200 ml of fresh blood throughout the peritoneal cavity, spreading to the subphrenic spaces (arrow). **B** Operative findings. Meckel’s diverticulum (arrow heads) was observed, with bleeding from surrounding mesentery (arrows)

An 11-year-old girl presented to another hospital with lower abdominal pain and vomiting that lasted for 2 days. Acute appendicitis was suspected, and she was referred to our department. On initial physical examination, her body temperature was 38.2 °C, and pulse and blood pressure were within normal ranges. Her abdomen was soft and mildly distended with tenderness localized to the lower abdomen. Laboratory data showed elevated levels of white blood cells (1.29 × 10^4^/μl) and C-reactive protein (3.69 mg/dl). Hemoglobin level was normal (14.1 g/dl). Abdominal enhanced computed tomography showed an abscess in the lower abdomen with ascites in the pelvis (Fig. 1). The patient was diagnosed with a localized intra-abdominal abscess and the decision was made to treat with antibiotics. However, her abdominal pain worsened, with abdominal distension, tenderness, and muscle guarding. She was diagnosed with panperitonitis and underwent surgery 5 h after admission. Laparoscopic observation from the umbilical region revealed 200 ml of fresh blood throughout the peritoneal cavity (Fig. 2A). The appendix looked normal, and the possibility of acute appendicitis was unlikely. Therefore, the umbilical incision was extended to identify a bleeding site, and an MD was detected associated with mesenteric bleeding (Fig. 2B). During surgery, the exact perforation point was undetectable. Small bowel resection was performed, and she was discharged without complication on the 5th postoperative day.

**Fig. 3:**
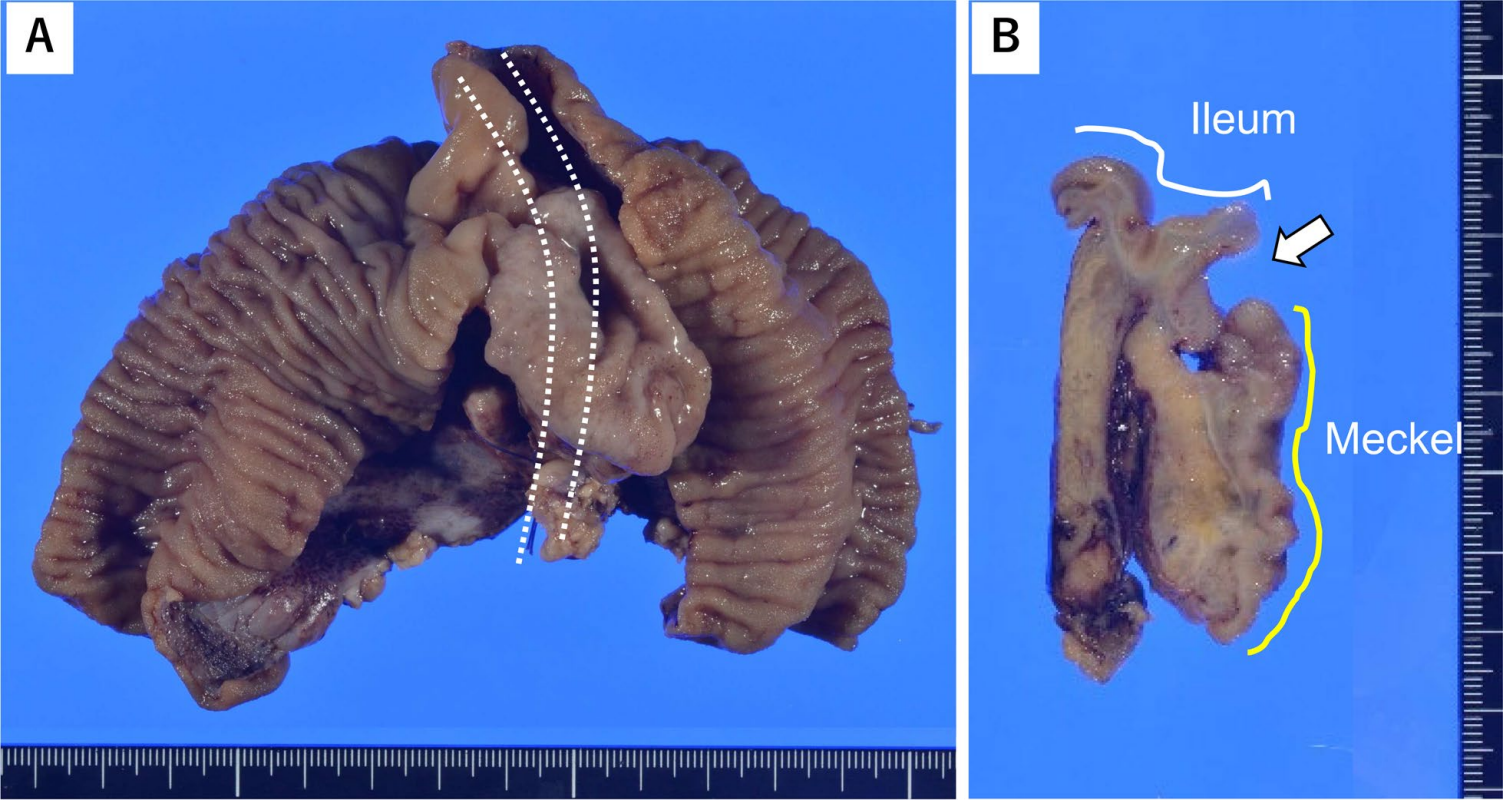
Macroscopic findings. **A** The Meckel’s diverticulum (MD), bulging in the small intestine, is indicated by white dotted lines. **B** Surface section of ileum-MD junction. A perforated lesion is indicated by an arrow between ileum (white line) and MD (yellow line)

**Fig. 4:**
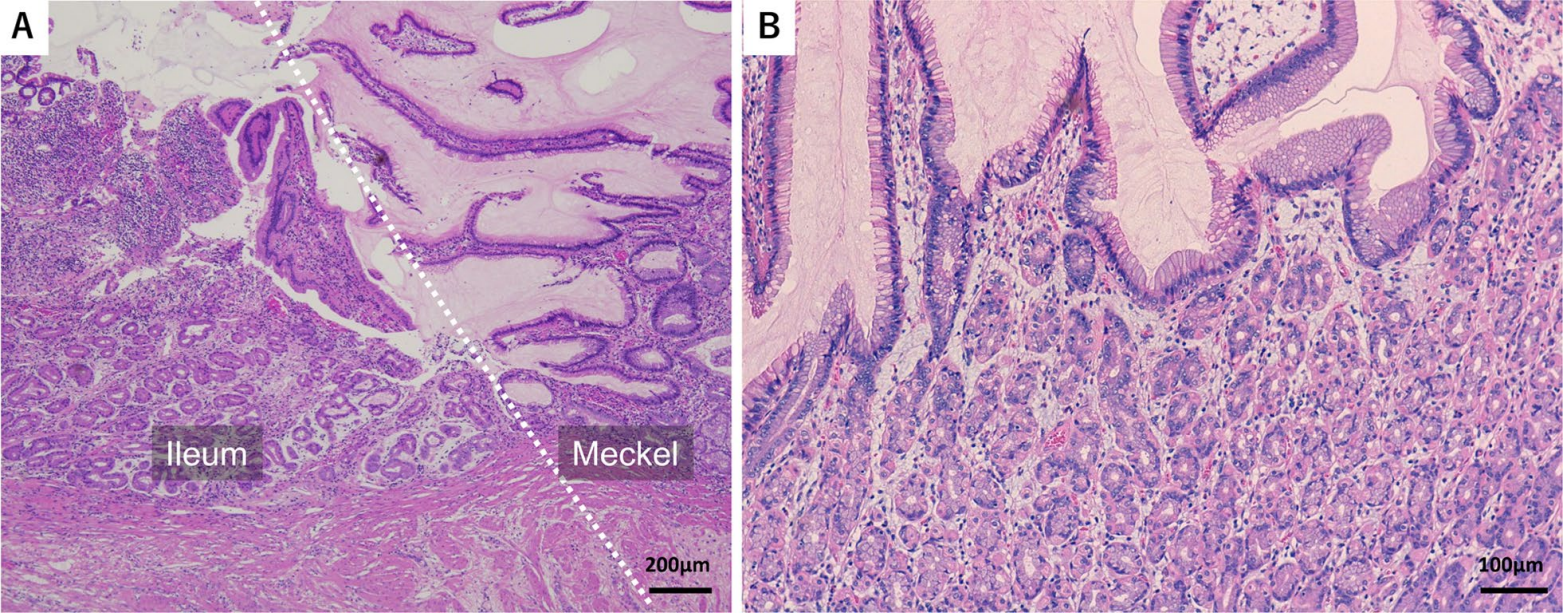
Microscopic findings (hematoxylin and eosin staining). **A** The ileum-Meckel’s diverticulum (MD) junction is indicated by a white dotted line. Surface epithelia of the ileum had erosion and inflammatory cells infiltration (upper left). Foveolar epithelia of MD were filled with gastric juice (upper right). **B** Ectopic gastric glands of the MD

Gross inspection of the resected specimen revealed an ileal perforation adjacent to MD junction (Fig. 3A, B). Microscopically, the ileum had a peptic ulcer that perforated muscular layer. The MD mucosa in the vicinity of the junction was composed of ectopic gastric glands, and foveolar epithelia were filled with gastric juice (Fig. 4A, B).

## Discussion

Symptomatic MD in children has been reported to present with obstruction in 46.7% of cases, gastrointestinal hemorrhage in 25.3%, and inflammation in 19.5% [1]. Intraperitoneal hemorrhage from MD is extremely rare in both adults and children. There have been only few reports of intraperitoneal hemorrhage because of MD, and only eight cases were given detailed information in PubMed (Table 1) [2–9]. Of the eight cases, three were children and five were adults. All were male, and the amount of blood loss ranged from 260 to 3000 ml. The MD was perforated in five cases and not perforated in three cases; ectopic gastric mucosa was found in the cases with perforation. Regarding the location and mechanism of bleeding, the causes of bleeding in MD can be classified into the following three categories: blood flow obstruction or inflammation (cases 1, 3, and 7); perforation with ectopic gastric mucosa (cases 2, 5 and 8); and other causes (cases 4 and 6). All cases resulting from blood flow obstruction or inflammation were adults, with or without perforation, and blood loss tended to be high. The pediatric cases (cases 2, 5 and 8) were the result of perforation with the presence of ectopic gastric mucosa. Other causes were blunt abdominal trauma caused by seat belts in a traffic accident and aneurysmal rupture of a mesodiverticular band to an MD.

**Table 1:** Previous reports of Meckel’s diverticulum with intraperitoneal hemorrhage

Case	Author	Year	Age/Sex	Blood loss	Red blood cell transfusion	Surgical findings and bleeding site	Status of MD	Perforation	Ectopic tissue
1	Sitaram et al. [2]	1991	34/M	Unknown	Unknown	MD with congestion and bleeding from the tip of the MD	Necrotic changes at the tip with ulceration of mucosa	No	No
2	Jelenc et al. [3]	2002	3/M	300 ml	Yes	MD with a perforation near the base and bleeding from there	Inflammation	Yes	Gastric mucosa
3	Burt et al. [4]	2006	63/M	1000 ml	1 unit	Bleeding from the distal portion of the MD with inflammation	Inflammation	No	No
4	Kazemi et al. [5]	2008	36/M	700 ml	No	Mesodiverticular rupture due to blunt abdominal trauma	Intact	Yes	Gastric mucosa
5	Borowski et al. [6]	2010	5/M	260 ml	No	Bleeding from the mucosal surface of a perforated MD	Inflammation	Yes	Gastric mucosa
6	Sommerhalder et al. [7]	2015	51/M	2000 ml	3 units	Aneurysmal rupture of a mesodiverticular band to an intact MD	Intact	No	Unknown
7	Rosat et al. [8]	2016	82/M	3000 ml	Unknown	Torsionated and perforated MD with intradiverticular bleeding	Strangulation by torsion	Yes	Unknown
8	Held et al. [9]	2018	10/M	Unknown	Unknown	Perforated MD eroded the adjacent mesentery resulting in bleeding	inflammation	Yes	Gastric mucosa
9	Our case	2021	11/F	200 ml	No	MD with bleeding from surrounding mesentery	Intact	Yes	Gastric mucosa

*MD* Meckel’s diverticulum; *M* male; *F* female

It is known that there is no difference in the incidence of MD between male and female, but most patients with symptomatic MD are male, and the male:female ratio is reported to be 1.5:1 to 4:1 [1]. There is a report that male is more likely to have ectopic tissue than female [10]. In addition, it is known that the presence of ectopic tissue increases the frequency of symptomatic MD [1]. Therefore, the higher frequency of ectopic tissue in male than female may be associated with more symptomatic MD in male than in female. In the above investigation, all cases of MD with intraperitoneal hemorrhage were male, whereas our case was female, which seems to be rare. Furthermore, in the above investigation, the mechanism of bleeding in pediatric cases was considered to be mainly perforation associated with ectopic gastric mucosa, which applies to our case.

It is not fully understood how MD causes intraperitoneal hemorrhage. In addition to specific causes, such as trauma and aneurysm rupture, there are two possible causes of intraperitoneal hemorrhage in MD: blood flow obstruction or inflammation, and perforation with ectopic gastric mucosa. Regarding blood flow obstruction or inflammation, it is assumed that intraperitoneal hemorrhage occurs because of the collapse of vessels in the submucosa and serosa. It is also known that gastrointestinal hemorrhage in MD can be caused by gastric acid from the ectopic gastric mucosa, resulting in small intestine ulceration [1]. Perforation with ectopic gastric mucosa is thought to be caused by bleeding into the abdominal cavity because of perforation by the same mechanism. In our case, the perforation was already closed, so it is unknown whether there was bleeding from it, but no bleeding was observed from the perforation site at the time of surgery. However, bleeding was observed from the mesenteric blood vessels that had adhered to the perforation site. This bleeding mechanism was similar to case 8 in Table 1 [9]. This may suggest that gastric acid had leaked into the abdominal cavity due to perforation disrupted mesenteric blood vessels, causing intraperitoneal hemorrhage.

## Conclusion

We report an extremely rare case of MD with intraperitoneal hemorrhage in a child. In pediatric cases, it is possible that perforation with ectopic gastric mucosa may cause massive bleeding because of rupture of the surrounding mesenteric blood vessels.

## Data Availability

All the data generated during this study are included in this published article.

## Abbreviation

MD: Meckel’s diverticulum
